# Infrared thermography in clinically healthy dogs shows body region and haircoat variability

**DOI:** 10.3389/fvets.2026.1868785

**Published:** 2026-07-28

**Authors:** Nathan Hall, Amber Hall, Lainey Atwood, Jessica Petry, Melissa J. Lewis

**Affiliations:** Department of Clinical Sciences, North Carolina State University College of Veterinary Medicine, Raleigh, NC, United States

**Keywords:** body surface temperature, canine, imaging, non-invasive, symmetry, thermal

## Abstract

Infrared thermography quantifies body surface temperature with growing applications in dogs, particularly among rehabilitation practitioners; however, incomplete understanding of normal temperature variation in healthy dogs limits interpretation. This study characterized body surface temperature distribution and evaluated factors influencing thermographic measurements in a heterogeneous group of healthy dogs. Client-owned, adult dogs were prospectively enrolled and underwent standardized thermographic imaging, with regions of interest defined for trunk, neck, and spine. Mean surface temperatures were assessed for symmetry, compared across regions and explored for relationships with individual dog characteristics. *p* < 0.05 was significant with adjustment for multiple comparisons. Thirty-three dogs were included with median age of 3.3 (1.0–10.9) years and median body weight of 18.6 (3.7–59.4) kgs. No significant left–right asymmetry was identified in paired regions. Mean (± SD) surface temperature for the dorsal neck (23.9° ± 1.6°) was significantly cooler than the lumbar spine (25.4° ± 1.9°, *p* = 0.009) and lateral trunk (25.2° ± 2.0°, *p* = 0.03) regions. Short-haired dogs exhibited significantly higher values than medium/long-haired dogs for the lateral trunk (26.8° ± 1.9° vs. 24.2° ± 1.4°, *p* = 0.001), dorsal neck (25.2° ± 1.8° vs. 23.2° ± 0.9°, *p* = 0.01) and sacral (26.0° ± 2.2 °C vs. 23.5° ± 1.1 °C, *p* = 0.006) regions. No other significant relationships were identified. Regional variation and coat length are important factors influencing thermography in healthy dogs, while bilateral symmetry is generally preserved. Establishing variability in healthy dogs is critical to clinical interpretation of thermography in abnormal dogs.

## 1 Introduction

Infrared thermography (IRT) is a non-invasive imaging modality that measures surface temperature by detecting infrared radiation emitted from the body (1–3). Because changes in blood flow, inflammation, and metabolic activity associated with various disease states can alter regional heat distribution (4, 5), IRT has gained interest as a diagnostic and monitoring tool in both human and veterinary medicine (2, 5, 6), with growing utilization among small animal rehabilitation practitioners. IRT has been explored in dogs and cats for applications including detection of musculoskeletal injury, inflammation, infection, neoplasia, spinal cord injury/disc herniation and evaluation of pain (7–10). The ability to detect localized changes in surface temperature may provide a non-invasive method for identifying abnormal regions and monitoring disease progression or response to treatment.

In veterinary patients, IRT is particularly attractive because it allows for evaluation of physiological changes without physical contact, sedation, or restraint (2, 3). Despite advantages and wide-ranging applications, several challenges limit the widespread clinical adoption of IRT in veterinary medicine. Main limitations include the lack of standardized baseline temperature data and variability in healthy animals (3). Surface temperature has been suggested to be influenced by numerous factors including patient characteristics, environmental and technical variables (1, 3, 4, 6, 11, 12, 30), but further characterization of these and other variables in dogs is warranted. Additionally, bilateral symmetry in temperature distribution is often assumed with asymmetry presumed to indicate localized pathology (6). However, the degree of natural variation in symmetry and across different body regions remains incompletely characterized in dogs. An improved understanding of normal variability is crucial to determining whether thermal differences represent pathological changes or natural variation. Establishing this information is of particular importance to rehabilitation practitioners utilizing IRT as part of their evaluation and management of dogs with various musculoskeletal and neurologic conditions.

This study characterized body surface temperature in a heterogeneous population of healthy adult dogs using a standardized IRT imaging protocol. Additionally, this study explored whether anatomic region, laterality or individual dog characteristics were associated with differences in thermographic measurements.

## 2 Materials and methods

### 2.1 Study design and population

This prospective study enrolled client-owned dogs recruited from students and staff at the NC State University College of Veterinary Medicine as well as dogs presented to the Primary Care Service for wellness visits. Dogs were considered eligible for inclusion if they were at least one year of age and apparently healthy based on unremarkable physical exam and no owner-reported health issues. Dogs presenting with overt illness or conditions that could influence thermographic measurements were excluded. All owners provided informed consent and this study was approved by the Institutional Animal Care and Use Committee (protocol # 24–162).

### 2.2 Clinical assessment

Prior to imaging, each dog underwent a physical examination including measuring core body temperature to confirm that they appeared clinically healthy at the time of participation. For each dog, the following information was recorded: age, sex, body weight, body condition score (BCS, 1–9) (13), haircoat type, haircoat color and temperament. Haircoat type was categorized as short or medium/long. Haircoat color was categorized as light, dark or spotted. Behavioral state was assigned using the Fear, Anxiety, and Stress (FAS) scoring system ranging from 0 to 4 (29). Since most dogs were rated as FAS 0 or 1, temperament was subsequently categorized as calm or anxious/excitable. Animals with minimal observable stress responses were categorized as calm, while those demonstrating greater signs of anxiety or excitability were categorized as anxious or excitable. Dogs that received medication prior to veterinary visits (e.g., trazodone) were not excluded but medication details were recorded.

### 2.3 Imaging acquisition

All dogs were imaged in a single, interior room without windows that was maintained at a steady ambient temperature of 21.7 °C for all imaging sessions. Prior to imaging, collars and harnesses were removed and there was a ten-minute acclimation period where the dog remained in the imaging area without direct contact or manipulation by investigators.

Thermographic images were obtained using a WellVu IR 640 medical-grade infrared thermal camera. The camera lens was positioned perpendicular to the dog’s body surface and adjusted to be approximately even with the height of the animal. Dogs were positioned standing squarely in a natural stance on a yoga mat placed in front of a matte black background. Handlers wore two pairs of nitrile examination gloves but avoided directly touching the dogs and occasionally used slip leashes as needed to aid in non-contact positioning. A standardized series of images was captured for each subject: left lateral, right lateral, dorsal, and ventral views. The dorsal views captured the neck to the tail, while the ventral view focused on the ventral cervical region. Images were captured in grayscale mode, with the body area of interest occupying the majority of the camera frame. Care was taken to maintain consistent camera positioning and distance between the camera and the dog. Each dog had a single imaging session.

### 2.4 Image processing

**Figure 1:**
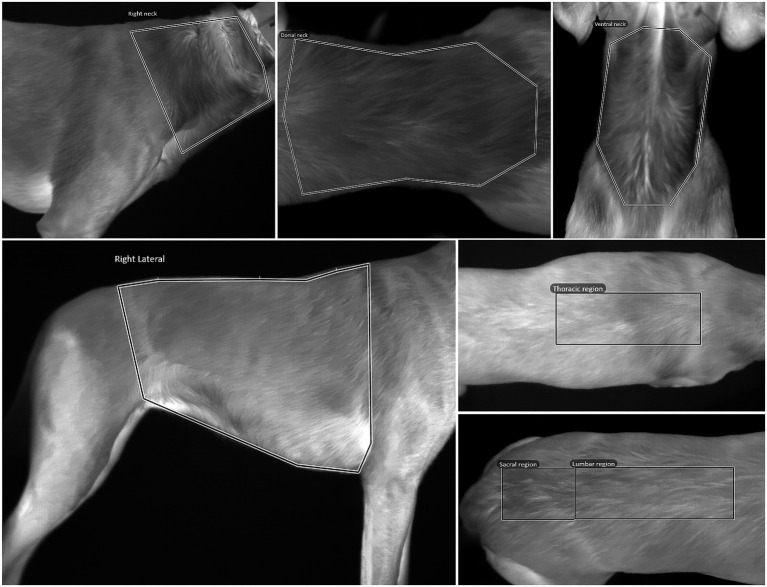
Representative examples of the regions of interest.

WellVu analysis software was used to manually create 9 regions of interest (ROIs) on the images using anatomical landmarks to ensure consistency across dogs (Figure 1). The following ROIs were included: left and right lateral trunk (thorax plus abdomen), left, right, dorsal and ventral neck, and the vertebral column subdivided into thoracic, lumbar and sacral regions. For each dog, WellVu software automatically calculated mean surface temperature (in °C) for each ROI.

### 2.5 Data analysis

Summary statistics were reported as mean (±SD) or median (range), as appropriate, and normality was assessed using the Shapiro–Wilk test. Mean temperature values for the individual dogs were averaged across dogs to produce mean values for each ROI, which were then used for analyses. To evaluate for asymmetry, mean temperature values for left versus right lateral trunk and lateral neck and dorsal versus ventral neck ROIs were compared using Student’s t-tests or Wilcoxon signed-rank tests as indicated. Comparisons of mean temperature across anatomical regions (lateral trunk, lateral neck, dorsal neck, ventral neck, thoracic, lumbar, and sacral regions) were performed using pairwise Student’s t-tests or Wilcoxon rank-sum tests, as appropriate. Associations between mean temperature and patient factors including age, sex, body weight, body condition score, coat type, coat color, and temperament were evaluated using simple linear regression, Student’s t-tests, or Wilcoxon rank-sum tests, as appropriate. These analyses were performed separately for the combined lateral trunk, dorsal neck and sacral values, regions chosen to explore potential associations across a range of mean temperatures. Analyses were performed using statistical software (Jmp student edition 18.2.0) and statistical significance was defined as *p* < 0.05. The Holm-Bonferroni method was used to correct for multiple comparisons, specifically when analyzing temperature differences between anatomical regions and when evaluating relationships to patient characteristics and reported as adjusted *p*-values. As this was a pilot study, power analysis was not performed, and results should be considered exploratory.

## 3 Results

### 3.1 Study population

Thirty-three dogs were enrolled with a median age of 3.3 years (1.0–10.9 years). There was 1 intact female, 19 spayed females, 2 intact males and 11 neutered males. Median body weight was 18.6 kgs (3.7–59.4 kgs). Median BCS was 5 (4–7). Mean core body temperature was 38.2° (± 0.65°) and was considered within normal limits for all dogs. Temperament was categorized as calm in 21 dogs and anxious/excitable in 12 dogs. One dog (classified as anxious) received trazodone the day of imaging. Haircoat type was classified as short in 12 dogs and medium/long dogs in 21 dogs. Haircoat color was categorized as dark in 17 dogs, light in 11 dogs, and spotted in 5 dogs. Dogs represented a variety of breeds, with the most common being mixed breed (*n* = 6), beagle (*n* = 4), and labrador retriever or labrador retriever mix (*n* = 4), with all other breeds represented by three or fewer individuals.

### 3.2 Imaging overview and evaluation of symmetry

**Figure 2:**
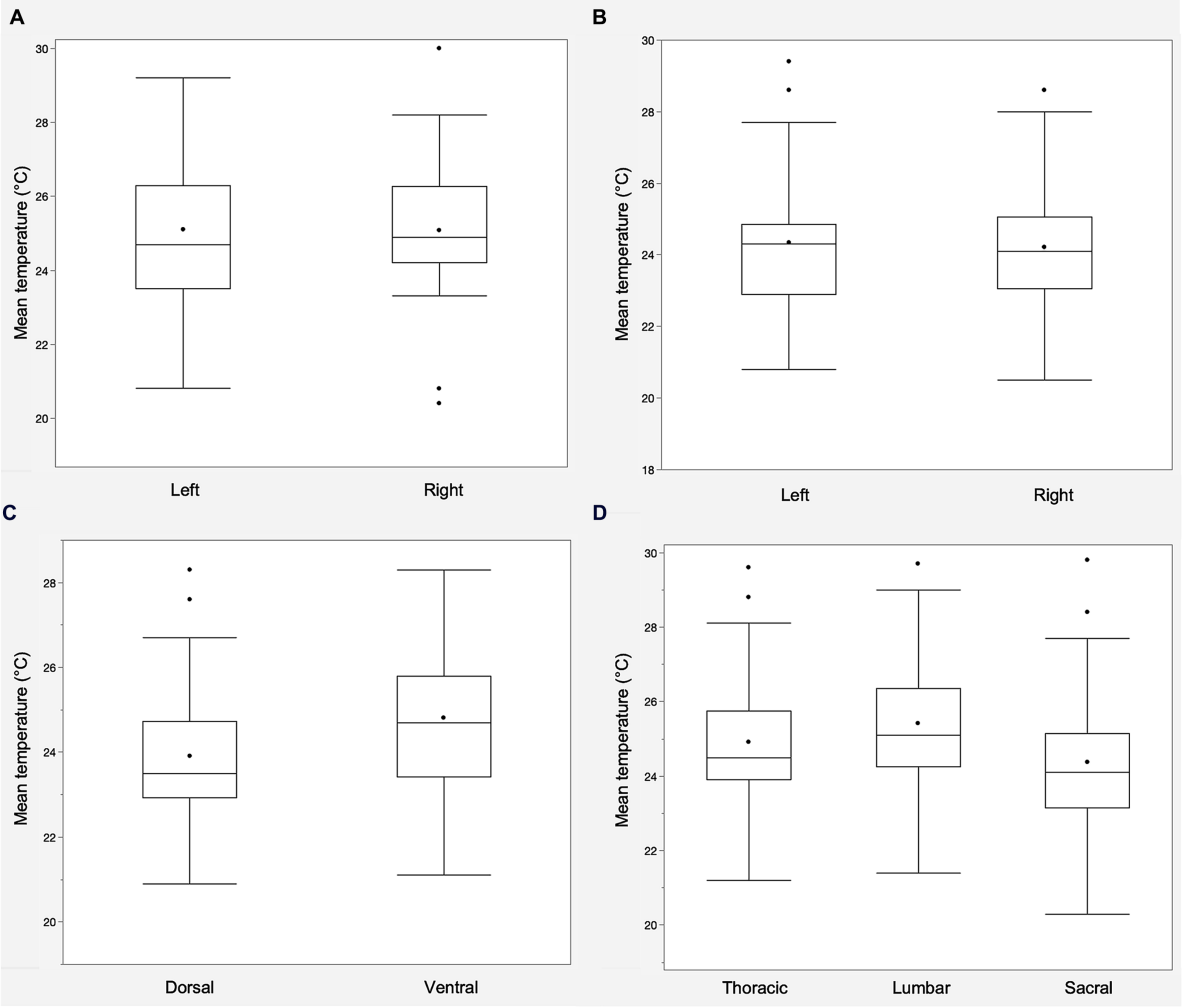
Boxplots showing summary and distribution of mean surface temperature (°C) in clinically healthy dogs for **(A)** left and right lateral trunk, **(B)** left and right lateral neck **(C)** dorsal and ventral neck and **(D)** vertebral column regions. Ventral neck was significantly warmer than dorsal neck region (*p* = 0.03) and lumbar was significantly warmer than sacral region (*p* = 0.04). The bottom and top of each box represent the first and third quartiles, respectively, the horizontal line within the box is the median, the point within the box is the mean, whiskers extend to 1.5 the interquartile range with data points beyond that depicted as individual points.

All dogs participated in image acquisition uneventfully. The pre-planned ROIs could be generated from the images for all dogs other than a single right lateral view that was missing in one dog. Summary statistics for all ROIs are presented in Table 1. Mean left lateral trunk temperature did not differ from right lateral trunk temperature (*p* = 0.97) (Figure 2A). Mean left lateral neck temperature did not differ from right lateral neck temperature (*p* = 0.80) (Figure 2B). Mean ventral neck temperature was higher than dorsal neck temperature (*p* = 0.03) (Figure 2C). Based on the absence of significant left–right differences, left and right lateral trunk and lateral neck ROIs were combined for subsequent analyses. Dorsal and ventral neck regions were maintained as separate variables for all analyses.

**Table 1:** Summary of body surface temperature (°C) for each body region.

Body region	Mean (°C)	SD	Minimum (°C)	Maximum (°C)
Left lateral trunk	25.1	2.1	20.8	29.2
Right lateral trunk	25.1	1.9	20.4	30.0
Combined lateral trunk	25.2	2.0	20.6	29.6
Left lateral neck	24.3	2.0	20.8	29.4
Right lateral neck	24.2	1.8	20.5	28.6
Combined lateral neck	24.4	1.9	20.7	29.0
Dorsal neck	23.9	1.6	20.9	28.3
Ventral neck	24.8	1.8	21.1	28.3
Thoracic	24.9	1.8	21.2	29.6
Lumbar	25.4	1.9	21.4	29.7
Sacral	24.4	2.0	20.3	29.8

### 3.3 Imaging across body regions

**Figure 3:**
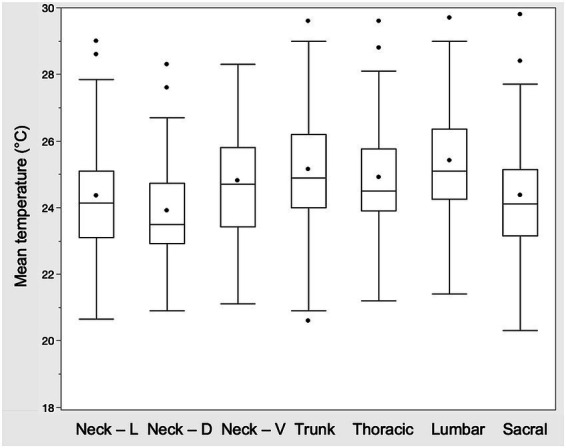
Boxplots showing summary and distribution of mean surface temperature (°C) in clinically healthy dogs across all anatomical regions evaluated. L, lateral; D, dorsal; V, ventral. The bottom and top of each box represent the first and third quartiles, respectively, the horizontal line within the box is the median, the point within the box is the mean, whiskers extend to 1.5 the interquartile range with data points beyond that depicted as individual points.

Mean temperatures differed across spinal regions with lumbar being the highest. Lumbar temperature was significantly higher than sacral temperature (*p* = 0.04), while thoracic vs. lumbar (*p* = 0.32) and thoracic vs. sacral (*p* = 0.32) comparisons were not significant (Figure 2D). Comparisons across all evaluated regions (combined lateral trunk, combined lateral neck, dorsal neck, ventral neck, thoracic spine, lumbar spine, and sacral spine) demonstrated significant variation in surface temperature (Supplementary material 1; Figure 3). Dorsal neck was the coolest region and lumbar spine was the warmest area followed by the lateral trunk. After adjusting for multiple comparisons, dorsal neck remained significantly cooler than the lumbar spine temperature (*p* = 0.009) and the combined lateral trunk temperature (*p* = 0.03).

### 3.4 Imaging relationships to dog characteristics

Lateral trunk temperature was not significantly associated with age, body weight, BCS or sex (*p* > 0.75, all comparisons). Mean trunk temperature was 25.1° ± 2.0° for female (spayed and intact) dogs and 25.2° ± 2.0° for male (neutered and intact) dogs. When the 3 intact animals (1 male and 2 females) were excluded, results remained unchanged (*p* = 1.00). Trunk temperatures were higher in anxious/excitable dogs (26.0° ± 1.7°) compared to calm dogs (24.7° ± 2.0°), although this difference was not statistically significant (*p* = 0.40). Dogs with short hair had significantly higher mean trunk temperatures (26.8° ± 1.9°) compared to dogs with medium/long hair (24.2° ± 1.4°, *p* = 0.001). Coat color was not associated with trunk temperature. Mean temperatures were 25.4° ± 1.9° for dark dogs, 25.0° ± 1.9° for light dogs, and 24.7° ± 1.2° for spotted dogs (*p* = 0.76). Excluding the 5 spotted dogs yielded similar results (*p* = 0.98). Dogs with short hair had significantly higher mean trunk temperatures (26.8° ± 1.9°) compared to dogs with medium/long hair (24.2° ± 1.4°, p = 0.001).

For the dorsal neck region, temperature was not associated with age, sex, body weight, BCS, temperament or coat color (*p* > 0.53, all comparisons). Short-haired dogs had significantly higher mean dorsal neck temperatures (25.2° ± 1.8°) compared to dogs with medium/long hair (23.2° ± 0.9°) (*p* = 0.01).

For the sacral region, temperature was not associated with age, sex, body weight, BCS, temperament or coat color (*p* > 0.18, all comparisons). Sacral temperatures differed significantly between dogs with short hair (26.0° ± 2.2°) compared to dogs with medium/long hair (23.5° ± 1.1°) (*p* = 0.006).

## 4 Discussion

In a heterogeneous group of healthy dogs, quantification of body surface temperature using IRT demonstrated minimal left to right asymmetry but did vary between different anatomical regions of the body and by length of haircoat. These findings provide a framework for important considerations when interpreting thermal images in both healthy and abnormal dogs.

No overt asymmetry was identified between left and right paired regions for the trunk or neck in this cohort of dogs. This finding is consistent with prior reports in healthy dogs where temperatures were comparable between left and right limbs or body (1, 6, 14). While some variation and minor asymmetry exists within individuals, our results provide additional support that it is reasonable to use the contralateral side as a reference standard, with more marked asymmetry in temperature likely to reflect localized pathology. This has been shown in dogs with unilateral lameness, where asymmetry in thermal images corresponds to asymmetry identified on force plate analysis (14). IRT has also been shown to be reasonably effective at differentiating normal versus abnormal limbs or joints within and across dogs (10, 15, 16). Additionally, in dogs presented with non-lateralized or generalized conditions (e.g., non-specific or poorly localized pain) for which IRT of the neck or trunk might be useful, our results suggest that consideration of left versus right is likely of minimal importance compared to other potential sources of variability.

Across anatomical regions, significant variation in surface temperature was observed with the warmest areas being the trunk and lumbar spine while the dorsal neck temperatures were consistently the coolest. Body surface temperature is influenced by vascular supply, tissue composition, and hair coverage and lack of uniformity of these features drives variation in thermal patterns across the body (4, 6). The relatively higher temperatures observed in the trunk and lumbar regions likely reflect greater muscle mass, vascular perfusion, and proximity to core body structures. In contrast, the dorsal neck may appear cooler due to thicker regional haircoat and greater distance from the core. These findings are consistent with prior studies demonstrating non-uniform thermal distribution, with warmer core-localized or thinner haircoat regions and cooler peripheral areas (17–20). These findings support that interpretation should be region-specific and that direct comparisons across regions, even adjacent areas or views, may be inappropriate without accounting for inherent physiological differences.

Haircoat type was the most consistent factor influencing IRT with short-haired dogs demonstrating significantly higher surface temperatures compared to medium- to long-haired dogs across multiple body regions. Hair coat characteristics have previously been identified as an important contributor to variability in IRT, as hair length, density, and texture can influence heat dissipation from the skin surface (1, 3, 6). In short-coated animals, there is likely reduced insulation, whereas longer or denser coats may attenuate the detectable infrared signal (1). Similarly, shaving the hair represents a more extreme variation in coat length and results in warmer temperatures than an intact coat (6). These findings reinforce the importance of accounting for coat length when interpreting thermographic images.

Other patient-level variables, including age, sex, body weight, and BCS, were not significantly associated with surface temperature. Age has been suggested in people to impact ocular temperatures measured with IRT (21). While age did not have an independent effect in this cohort, age-related changes in vascular function or tissue composition could still influence regional heat distribution. Given this, age may remain a relevant variable to consider in larger or more age-diverse populations. Body weight and BCS were not associated with temperature. Body size and adiposity have been shown to influence thermoregulation through insulation and surface area effects in dogs (22, 23). However, two thirds of the dogs were classified as 5/9, potentially limiting the ability to detect meaningful differences related to body composition in this cohort. The small number of intact animals limited assessment of sex and hormonal effects. Hormonal influences on IRT have not been clearly demonstrated in people (24) or intact female dogs (25), but body surface temperature differs between men and women, depending on the body region (26, 27). This is speculated to be due to sex-based differences in metabolic rate, peripheral blood flow and body mass (26, 27), but this has not been specifically evaluated in dogs.

No significant differences were documented based on temperament, comparing anxious or excitable dogs to calm dogs. However, it remains possible that behavioral factors such as temperament and stress level could influence surface temperature. This has been speculated to occur through activation of the sympathetic nervous system, which can alter peripheral blood flow and thermal distribution patterns (2, 5). Response to painful or stressful events has been evaluated in dogs using IRT of the face or eyes (4, 28), but the effect of emotional state on other areas of the body might be worthy of further exploration. Since dogs that are anxious or excitable during examination may exhibit physiological responses that influence thermographic readings, incorporation of validated, objective measures of behavioral state might be important.

This study is limited by small group size and analyses should be considered exploratory. While intentionally heterogeneous, there was limited ability to assess the influence of some aspects such as breed, age and BCS. Behavioral factors, including stress level and response to the acclimation period, could not be fully standardized and may have contributed to observed variability. Additionally, dogs were judged to be clinically healthy based on owner testament and unremarkable physical examinations, but it is possible less obvious abnormalities could have been present and influenced results. Our imaging protocol also captured all dogs at rest. Treadmill exercise in dogs has been demonstrated to impact body surface temperature and to vary by body region (20), but it remains unknown to what extent certain patient characteristics (e.g., hair coat type) influence body surface temperature for the exercising (or post-exercising) dog. The post-exercise thermal environment could be more thoroughly explored to optimize the potential applications for IRT in the rehabilitation setting. We relied on absolute temperature values rather than relative thermal patterns, but it is possible a combination will be most useful when using IRT as a screening tool in dogs.

Overall, our results in a group of healthy dogs provide an important step in identifying sources of variability in IRT. Significant regional variation, even among adjacent areas, emphasizes that such differences should not necessarily be interpreted to reflect a pathologic process. Continued exploration of these and other variables as sources of inter- and intra-individual temperature variability is warranted to optimize the clinical utility of IRT as a tool in dogs as part of comprehensive rehabilitation assessments.

## Data Availability

The raw data supporting the conclusions of this article will be made available by the authors, without undue reservation.
