# Supplementary material for: Infrared thermography in clinically healthy dogs shows body region and haircoat variability
Source: Front Vet Sci. 2026 Jul 28;13:1868785. doi: 10.3389/fvets.2026.1868785 (PMC13457002; doi:10.3389/fvets.2026.1868785)
Supplement: Supplementary file 1 [file Table_1.DOCX]

Supplementary Material 1. Pairwise comparisons of mean body surface temperature between anatomical regions in healthy dogs. * denotes statistical significance. P_a_: adjusted p-value.

| **Body Region** | **Body Region** | **p-value** | **p_a_-value** |
| --- | --- | --- | --- |
| Lumbar | Neck - dorsal | 0.0006* | 0.009* |
| Trunk | Neck - dorsal | 0.002* | 0.03* |
| Thoracic | Neck - dorsal | 0.01* | 0.13 |
| Sacral | Lumbar | 0.01* | 0.18 |
| Lumbar | Neck - lateral | 0.02* | 0.20 |
| Neck - dorsal | Neck - ventral | 0.03* | 0.30 |
| Trunk | Sacral | 0.04* | 0.37 |
| All other regional comparisons | | >0.06 | >0.48 |
